# The utility of intraoperative magnetic resonance imaging in recurrent glioblastoma surgery: A volumetric analysis

**DOI:** 10.1016/j.bas.2026.106135

**Published:** 2026-06-30

**Authors:** Obada T. Alhalabi, Kirill Mironov, Khurshed Nabiev, Ahmed Nor Ghraieb, Maximilian Jordan, Stefan Joser, Martin Bendszus, Sandro M. Krieg, Philip Dao Trong, Andreas W. Unterberg, Christine Jungk

**Affiliations:** a Department of Neurosurgery, Heidelberg University Hospital, Heidelberg, Germany; b Medical Faculty, University Heidelberg, Heidelberg, Germany; c Clinical Cooperation Unit Neurooncology, German Cancer Consortium (DKTK), German Cancer Research Center (DKFZ), Heidelberg, Germany; d Department of Neurosurgery, Vivantes Hospital Neu-Koeln, Berlin, Germany; e Department of Neuroradiology, University Hospital Heidelberg, Heidelberg, Germany

**Keywords:** Recurrent glioblastoma, Intraoperative magnetic resonance imaging, Volumetric analysis, Neurological morbidity, Post-surgery survival

## Abstract

**Objective:**

Intraoperative magnetic resonance imaging (iMRI) for the resection of newly diagnosed glioblastoma has shown efficacy comparable to fluorescence guidance; however, its role in recurrent glioblastoma (recGB) remains unclear. This study evaluated the efficacy and safety of iMRI-guided recGB surgery.

**Methods:**

Consecutive patients with recGB undergoing iMRI-guided re-resection between 2015 and 2023 were retrospectively analyzed. Contrast-enhancing (CE) tumor volumes from preoperative (3T), intraoperative (1.5T), and early postoperative (epMRI, 3T) scans were assessed per RANO Resect criteria. The impact of iMRI on intraoperative decision-making for additional resection (AR), functional outcomes, and survival was evaluated.

**Results:**

A total of 150 patients were included. Complete CE tumor resection was intended in 85% (n = 127). iMRI prompted AR in 77% (n = 115), increasing RANO class 1–2 resections from 58% on iMRI to 85% on epMRI. A “RANO-switch” (conversion from class 3 on iMRI to class 1–2 on epMRI) occurred in 27% (n = 41). Preoperative CE tumor volume was the only independent predictor of AR (p = 0.001), with 6.9 ml identified as the cut-off above which AR was significantly more likely to achieve a “RANO-switch.” Neurological deterioration was not associated with AR (transient: 15% vs 16%; permanent: 2% vs 3%). Median survival after re-resection was 11 months. Gross total resection (residual tumor volume ≤0.175 ml), but not RANO class, independently predicted improved survival (HR 0.50, p = 0.006).

**Conclusion:**

In recGB, where a real-time intraoperative information about RTV could be relevant, iMRI significantly improved the extent of resection without increasing the risk of neurological deficits. Larger preoperative CE tumor volumes were associated with a greater resection benefit from iMRI, supporting its efficacy and safety in recGB surgery.

## Highlights

• iMRI increased complete resections in recurrent glioblastoma without added risk.
• Larger preoperative tumor volumes predicted greater benefit from iMRI guidance.
• Gross total resection independently improved survival outcomes in recGB.

**Key points**

• iMRI increased complete resections in recurrent glioblastoma without added risk of neurological deficits.
• Larger preoperative tumor volumes predicted greater benefit from iMRI guidance.
• Gross total resection independently improved survival outcomes in recGB.

**Impact of the study**

The role of intraoperative MRI (iMRI) in recurrent glioblastoma (recGB) surgery has been unclear, as most evidence focuses on its use in newly diagnosed cases. This study demonstrates that iMRI substantially increases the extent of resection in recGB without compromising neurological safety, leading to more oncologically meaningful resections. Importantly, patients with larger preoperative contrast-enhancing tumor volumes derived the greatest benefit, identifying a subgroup particularly suited for iMRI-guided surgery. Moreover, gross total resection—but not RANO classification—was independently associated with improved survival after re-resection, underscoring the prognostic value of achieving minimal residual tumor volume. These findings provide strong clinical evidence supporting the routine integration of iMRI in the surgical management of recGB, where residual tumor volume is of paramount importance, and may inform patient selection and surgical planning to optimize outcomes.

## 1 Introduction

Glioblastoma (GB) is an aggressive, therapy-resistant intrinsic brain tumor characterized by isocitrate dehydrogenase (IDH) wildtype status and poor prognosis (Stupp et al., 2005; Wen et al., 2020). Despite multimodal therapy, tumor recurrence is almost inevitable, with no consensus on the standard-of-care at relapse (van Opijnen et al., 2024). Several studies have emphasized the clinical benefit of re-resection for selected patients dependent on tumor location and functional status, particularly in relation to limited effective systemic therapies for recurrent glioblastoma (recGB) (Leone et al., 2022; Ringel et al., 2016; Weller et al., 2019).

The importance of maximizing the extent of resection (EOR) at re-resection has been well established (Suchorska et al., 2016; Uppstrom et al., 2016; Karschnia et al., 2023; Bloch et al., 2012), with median survival after re-resection reported at 12 to 18 months (Alhalabi et al., 2024; Yang et al., 2022; Woo et al., 2024). The prognostic significance of EOR has also been quantified by the Response Assessment in Neuro-Oncology (RANO) Resect group, which demonstrated in a multi-center study that a contrast-enhancing (CE) residual tumor volume (RTV) of ≤1 ml (RANO class 2) on early postoperative MRI (epMRI) was associated with improved survival, while supramarginal resection (RANO class 1) did not confer additional survival benefit over RANO class 2^9^.

In newly diagnosed GB, intraoperative MRI (iMRI) has shown superiority over conventional white-light surgery in achieving maximized resections (Senft et al., 2011; Zhang et al., 2023). Fluorescence-guided surgery using 5-aminolevulinic acid (5-ALA) has also been found superior to white-light surgery (Roder et al., 2023; Stummer et al., 2006) while showing comparable to iMRI. In a recent prospective controlled multicenter clinical trial, complete resection (CR) of the CE tumor was achieved in 78% of 5-ALA- and 81% of iMRI-guided surgeries (Roder et al., 2023).

In contrast, data evaluating the utility of iMRI-guided resections in recGB (Quick-Weller et al., 2016) is limited, especially with the increased importance of real-time information on RTV in eloquent recurrent GB. In this study, we investigated the role of iMRI in maximizing the EOR during re-resection and assessed its impact on postoperative neurological outcomes and survival. Additionally, we sought to determine predictive factors that identify patients most likely to benefit from iMRI-guidance at re-resection.

## 2 Methods

### 2.1 Study population

A consecutive cohort of patients with iMRI-guided re-resection of a previously resected IDH-wildtype glioblastoma (as per WHO 2021 classification (Weller et al., 2021)) treated between 2015 and 2023 at the Department of Neurosurgery, University Hospital Heidelberg was retrospectively identified from our institutional database. Only patients with unequivocal GB recurrence based on histopathologically diagnosed vital tumor were included. Cases with radiation-induced necrosis alone or in combination with vital tumor as per histopathological report as well as patients without early postoperative MRI (epMRI) were excluded. Availability of digitalized preoperative MRI (preopMRI, 3 T S Vario™, Prisma™ or Skyra™), iMRI (1.5T, Siemens Espree™), and epMRI (MRI Siemens as preopMRI, within 48 h) were a prerequisite for inclusion. Continuing tumor resection after iMRI is termed ‘additional resection’ (AR). Demographic and clinical data was extracted from medical charts.

### 2.2 Tumor volumetry

Volumetric analysis of CE tumor volumes on preoperative, intraoperative, and postoperative post-contrast T1-weighted MR was performed by semi-automated segmentation and measurement using the Brainlab™ software SmartBrush version 4.5 (Brainlab, Germany). The RANO Resect group criteria were utilized to classify patients into RANO classes 1 to 3^9^: RANO class 1: 0 ml CE tumor + ≤5 ml non-CE, FLAIR-hyperintense tumor, RANO class 2: 0–1 ml CE tumor ± >5 ml non-CE tumor. RANO class 3: >1 ml CE tumor. The term ‘RANO-switch’ refers to cases where iMRI showed a CE-RTV of >1 ml, equivalent to RANO class 3, which after AR, was reduced to <1 ml CE-RTV on epMRI. Tumors with anatomical localization within motor, sensory, language and visual cortical and subcortical areas as described by Chang et al. (2008), were defined as “eloquent”, in addition to deep-seated lesions (thalamic, basal ganglia).

### 2.3 Study endpoints

Patients were followed until death or loss to follow-up (censored on the day of last follow-up), with survival after re-resection defined as the interval from the date of re-resection to death from any cause or censorship. ‘Transient’ postoperative neurological deficits were defined as deficits resolving within 30 days, which otherwise are termed ‘permanent’.

### 2.4 Statistical analysis

Statistical analyses were performed using GraphPad PRISM (Version 10, GraphPad Software, Inc., La Jolla, USA) and SPSS Statistics (version 29.0.0.0, IBM Corp, Armonk, NY, USA). Continuous variables were reported as means ± standard deviation and/or median and interquartile range (IQR). Categorial variables were presented as numbers and percentages and compared using Chi-Square and Fisher's exact test. Binary logistic regression was used to identify factors predicting the likelihood of AR. To estimate the probability of AR dependent on the preoperative tumor volume, a quadratic logistic regression model was applied. For this, tumor volume was centered to improve numerical stability, and both linear and squared terms were included as predictors. To determine an optimal volume threshold, the Youden index was computed across all tumor volumes (maximizing the sum of sensitivity and specificity minus one). Survival analysis was performed by univariate Log-rank (Mantel-Cox) tests and a multivariable Cox Proportional Hazards regression model with stepwise forward selection of variables included based on univariate significance or clinical relevance. Significance was defined as p < 0.05.

## 3 Ethics approval

The Ethics Committee at the University of Heidelberg approved the protocol of this retrospective analysis under S-455/2023 which has been performed in accordance with the ethical standards laid down in the 1964 Declaration of Helsinki and its later amendments and for which patient consent was not required.

## 4 Results

### 4.1 Patient characteristics

We identified 211 recGB patients who underwent iMRI-guided resection. After exclusion of 55 cases with histopathologically confirmed radiation-induced necrosis, 129 (86%) patients at 1st recurrence and 21 (14%) patients at 2nd, 3rd, or 4th recurrence remained (Table 1). In 42 cases, intraoperative MRI was not performed and in 19 cases, early postoperative MRI was not performed for logistic reasons. The cohort comprised 94 males (63%) and 56 females (37%) with a median age of 58.4 years [IQR 14 years]. Tumor localization was mostly temporal (42%, n = 63) and frontal (28%, n = 43). Eloquent areas were involved in 45% (n = 68). Awake craniotomy was performed in 4 (3%) and intraoperative neuromonitoring (IONM) under general anesthesia in another 18 (12%) patients. In all cases, neuropathology reports confirmed vital tumor on resected specimens. *MGMT*-promotor was methylated in 43 (29%), unmethylated in 78 (52%) und unknown in 29 (19%) patients. Mean preoperative KPS was unchanged at discharge (83.0 ± 10.4 vs 81.0 ± 10.8; p = 0.055, Welch's *t*-test). In most cases (79%, n = 118), surgery was followed by chemotherapy, including temozolomide (TMZ) in 24 (16%), CCNU/VP-16 in 77 (51%) and BCNU/VM-26 in 9 cases (6%) (Table 1). Bevacizumab was applied in 15 (10%) and targeted therapies in 19 (13%) patients. 26 patients (17%) underwent postoperative radiotherapy. In 29 cases (19%), no adjuvant treatment was applied.

**Table 1:** **Patient characteristics of the iMRI cohort**. ^a^Other agents include carboplatin, hydroxycarbamid. abemaciclib. SD= Standard deviation. IQR= Inter-quartile Range. MGMT = O6-methylguanine-DNA methyltransferase. CCNU = Lomustine. VP-16 = Etoposide. BCNU= Carmustine. VM-26 = Teniposide.

Variable	RANO 1-2 (n = 86)	RANO “switch” (n = 41)	RANO 3 (n = 23)	p-value
Gender Male Female	51 (59%) 35 (41%)	31 (76%) 10 (24%)	12 (52%) 11 (48%)	0.109^a^
Age Mean (SD) Median (IQR)	57.3 ± 10.9 57.0 (14.5)	61.6 ± 9.2 62.0 (13.0)	57.5 ± 9.2 58.7 (11.3)	0.062^b^
Intraoperative neuromonitoring±awake craniotomy	10 (6.7%)	4 (7%)	4 (17%)	0.657^a^
MGMT promoter methylation status Methylated Non-methylated Unknown	27 (31%) 42 (49%) 17 (20%)	9 (24%) 24 (59%) 8 (17%)	7 (30%) 12 (52%) 4 (17%)	0.500^a^
Tumor localization Frontal Temporal Parietal Occipital Insular Deep	21 (27%) 43 (50%) 14 (16%) 8 (9%) 0 (0%) 0 (0%)	14 (34%) 14 (34%) 8 (20%) 4 (7%) 1 (2%) 0 (0%)	8 (33%) 6 (26%) 2 (9%) 4 (17%) 2 (9%) 1 (2%)	0.410^a^ 0.058^a^ 0.524^a^ 0.523^a^ **0.030^a^** 0.062^a^
Tumor eloquence	31 (36%)	20 (49%)	17 (74%)	**0.005^a^**
Stage of disease at the time of re-resection First recurrence Second recurrence Third recurrence Fourth recurrence	77 (90%) 5 (6%) 3 (3%) 1 (1%)	33 (81%) 7 (17%) 1 (2%) 0 (0%)	16 (70%) 6 (26%) 1 (4%) 0 (0%)	0.052^a^ 0.015^a^ 0.913^a^ 0.688^a^
Time between first and second resection (months) Mean ± SD	16.1 (13.2)	12.2 (7.1)	15.6 (13.3)	0.234^b^
Mean KPS before surgery	83.7	81.0	83.5	0.369^b^
Mean KPS at discharge	81.9	79.0	83.4	0.273^b^
Adjuvant therapy after re-resection Temozolomide CCNU/VP-16 BCNU/VM-26 Unknown Other agents: Bevacizumab Other^d^	13 (19%) 49 (57%) 3 (4%) 2 (6%) 7 (8%) 12 (14%)	9 (22%) 16 (39%) 3 (7%) 0 (0%) 6 (15%) 4 (10%)	2 (9%) 13 (57%) 2 (9%) 2 (9%) 1 (4%) 2 (9%)	0.251^c^ 0.360^a^ 0.149^a^ 0.493^a^ 0.112^a^ 0.336^a^ 0.689^a^
Radiotherapy	17 (20%)	7 (17%)	2 (9%)	0.460^a^
No adjuvant treatment	7 (8%)	5 (12%)	2 (9%)	0.759^a^
Median progression-free survival after first resection (months)	10	8	8.5	0.198^c^

### 4.2 Routine use of iMRI for recurrent glioblastoma surgery

**Fig. 1:**
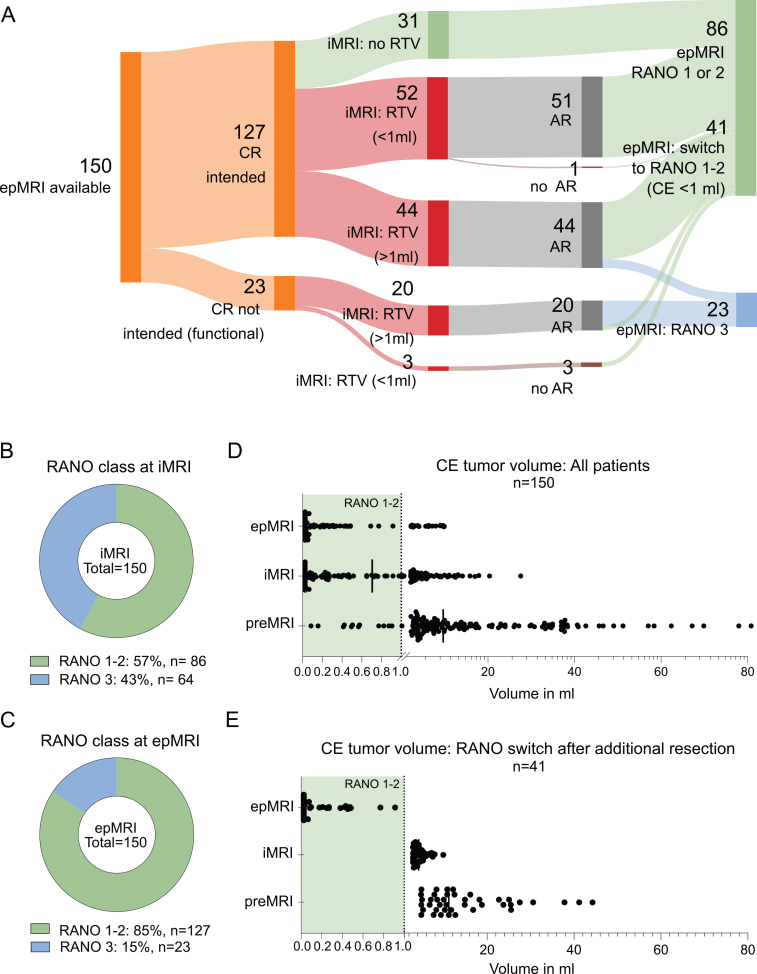
**Analysis of the efficacy of intraoperative MRI guidance in recurrent glioblastoma surgery. A:** Sankey plot depicting the fate of cases undergoing iMRI-guided re-resection with (n = 150) epMRI available; iMRI = intraoperative MRI; epMRI = early postoperative MRI; CR = complete resection; CE = contrast enhancing; RTV = residual tumor volume; AR = additional resection; RANO= Response Assessment in Neuro-Oncology (RANO) classification). **B:** Proportions of patients with their resection status classified by RANO Resect categories based on residual CE tumor volumes on iMRI, given for the final iMRI cohort (n = 150) **C**: CE tumor volumes determined on preoperative MRI (preopMRI), intraoperative MRI (iMRI), and early postoperative MRI (epMRI), stratified by the RANO Resect status at epMRI (RANO class 1 and 2 in green). Notice the “RANO-switch” of patients between iMRI and epMRI. **D:** Proportions of patients with their resection status classified by RANO resect categories based on residual CE tumor volumes on epMRI, given for the final iMRI cohort. Note the increase in RANO class 1 and 2 resections from iMRI to epMRI. **E:** CE tumor volumes measured on preopMRI, iMRI, epMRI in patients showing RANO class 3 at iMRI but RANO class 1-2 at epMRI after additional resection (“RANO-switch” group; n = 41).

Based on preopMRI, complete resection (CR) of the CE tumor was intended in 127 patients (85%). In the remaining 23 cases (15%), CR was not intended due to anticipated functional limitations. In patients with intended CR, iMRI showed no CE-RTV in 31 of 127 cases only (24%), which was confirmed on epMRI. For the remaining 96 patients, the RTV on iMRI was <1 ml in 52 cases (41%) and >1 ml in 44 cases (29%). Following iMRI, 95 of 127 patients (74.8%) with intended CR and 20 of 23 patients (87%) without intended CR underwent additional resection (AR) (Fig. 1A). Hence, the overall rate of AR was 77% (n = 115). Upon iMRI, a RANO class 1-2 resection (RTV ≤ 1 ml) was noted in 86 patients (57%) and a RANO class 3 resection (RTV > 1 ml) in 64 patients (43%) (Fig. 1B). After AR, epMRI revealed RANO class 1-2 resection in 127 patients (85%). A “RANO-switch”, i.e. conversion from RANO class 3 on iMRI to RANO class 1-2 on epMRI, was possible in 41 patients (27%). Accordingly, RANO class 3 resections were observed in 3 of 127 patients with intended CR (4%), and in 20 of 23 patients without intended CR (87%). Overall, the use of iMRI increased the proportion of patients with RANO class 1–2 from 57% on iMRI (n = 86; Fig. 1B) to 85% on epMRI (n = 127, Fig. 1C, Supplementary Fig. S1). In the complete cohort, the mean preoperative CE tumor volume was 15.4 ml (SD 16.7), which was reduced to 2.57 ml (SD 4.28 ml) on iMRI and 0.74 ml (SD 1.91) on epMRI (p = 0.001, one-way ANOVA; Fig. 1D). In “RANO-switch” patients (n = 41), the mean preoperative CE volume was 19.70 ml (SD 15.62), 3.56 ml (SD 2.54) on iMRI and 0.13 ml (SD 0.21) on epMRI (p = 0.001, one-way ANOVA, Fig. 1E–Table 2).

**Table 2:** **Comparative analysis of potential prognostic factors between different resection groups according to the results on epMRI**. Classification is based on the Response Assessment in Neuro-Oncology (RANO) classification. ^a^Chi-Square-Test. ^b^One-way Analysis of Variance (ANOVA). ^c^Logrank (Mantel-Cox) test. ^d^Other agents include carboplatin, hydroxycarbamid. abemaciclib. SD= Standard deviation. IQR= Inter-quartile Range. MGMT = O6-methylguanine-DNA methyltransferase. CCNU = Lomustine. VP-16 = Etoposide. BCNU= Carmustine. VM-26 = Teniposide. Significant comparisons are highlighted in bold.

Variable	β	p-value	OR	95% CI
Surgery at 1st recurrence (yes)	0.716	0.370	2.05	0.43-9.81
Sex (female)	0.085	0.857	1.09	0.43-2.73
Age at re-resection (years; cont.)	0.001	0.975	1.00	0.96-1.04
5-ALA (yes)	−0.705	0.651	0.49	0.02-10.50
IONM±Awake craniotomy (yes)	0.135	0.863	1.15	0.25-5.29
Tumor eloquence	0.637	0.268	1.89	0.61-5.83
Tumor localization				
- frontal vs. temporal	−0.333	0.541	0.72	0.25-2.09
- frontal vs. others	−0.085	0.895	0.92	0.26-3.25
Tumor lateralization (left)	−0.009	0.985	0.99	0.40-2.45
**Preoperative tumor volume (ml; cont.)**	**0.112**	**0.001**	**1.12**	**1.04**–**1.11**
CR intended (yes)	1.049	0.254	2.86	0.47-17.36
Neurological deficit pre-op (yes)	0.310	0.548	1.36	0.50-3.75
KPS ≥70 (yes)	0.338	0.784	1.40	0.13-15.7

### 4.3 Preoperative tumor volume predicts the likelihood of additional resection after iMRI

**Fig. 2:**
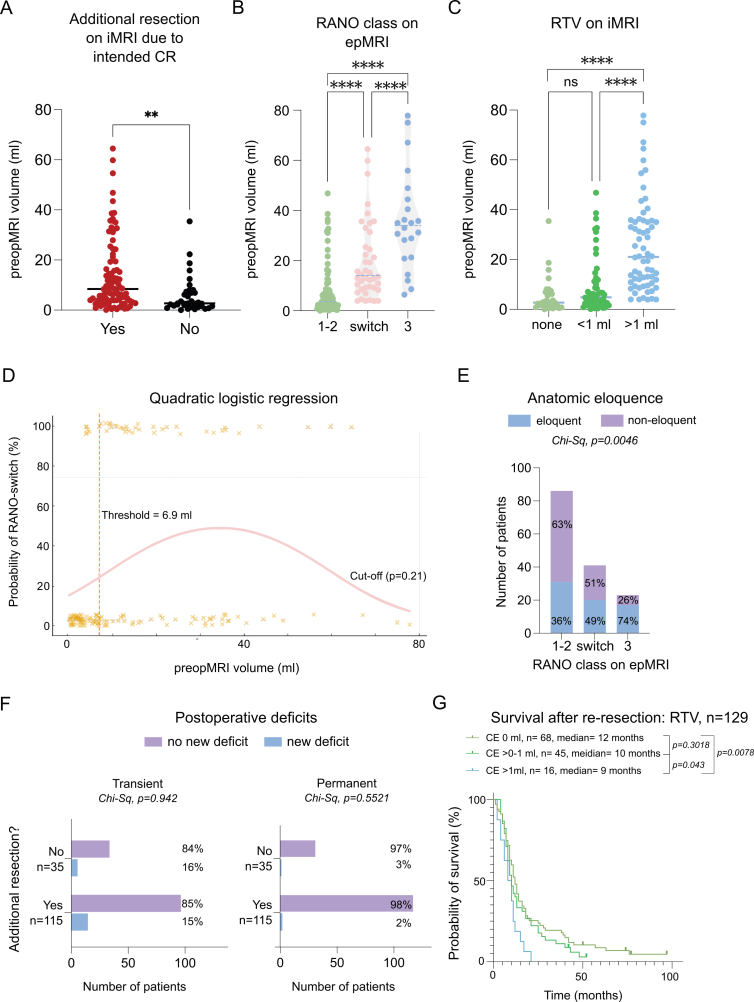
**Impact of preoperative tumor volumes on additional resection after intraoperative MRI along with volumetric, neurological and survival outcomes.** A: preopMRI CE tumor volumes in patients with intended CR, n = 127, stratified by whether an additional resection was performed or not (**p < 0.01, unpaired *t*-test). **B:** preopMRI CE tumor volumes in patients stratified by RANO Resect classes on epMRI. “switch” denotes showing RANO class 3 at iMRI but RANO class 1-2 at epMRI due to additional resection. **C:** preopMRI CE tumor volumes in patients stratified by prognostically relevant CE-RTV) thresholds on iMRI: none, <1 ml = potentially do not profit from additional resection, >1 ml = profit from additional resection). Analysis of Variance (ANOVA), **p < 0.01, ***p < 0.001, ****p < 0.0001, n = 150. **D:** Quadratic logistic regression model showing the predicted probability of RANO-Switch (Y = 1) as a function of preoperative tumor volume. The solid line represents the model-predicted probability, and the dashed vertical line indicates the optimal cut-off value (6.90 ml), above which a RANO-Switch is predicted. Individual patient observations are shown as orange crosses. Sensitivity ≈0.854, Specificity ≈0.569, Youden's J ≈ 0.422. **E:** Distribution of tumors involving eloquent versus non-eloquent brain regions in patients depending on RANO Resect categories (RANO 1 and 2 = 86 patients, switch = 41 patients, RANO 3 = 23 patients, Chi-Square-test = results embedded in figure. **F:** Distribution of postoperative deficits (transient or permanent) in patients who underwent additional resection after iMRI compared to those who did not. **G:** Kaplan-Meier survival analysis of patients stratified by CE-RTV at epMRI (0 ml, <1 ml, > 1 ml, Log-rank (Mantel-Cox) test with p-values embedded in figure).

We further investigated factors predicting the likelihood of AR, besides the surgeon's estimation of resectability. To this end, we performed a binary logistic regression analysis which revealed that only preoperative tumor volume was significantly associated with AR (β = 0.112, p = 0.001; OR 1.118, 95% CI 1.044–1.197; (Table 3 includes all covariates). Indeed, in cases with intended CR, mean preoperative tumor volumes were significantly higher in patients who underwent AR (13.92 ml; n = 96) compared to patients without AR (mean 5.82 ml; n = 31, p = 0.0019, unpaired *t*-test, Fig. 2A). Specifically, patients of the “RANO-switch” group had significantly larger preoperative volumes (mean 19.7 ml, SD 15.62) compared to those who already achieved RANO 1–2 status on iMRI (mean 7.87 ml, SD 10.16; p < 0.001, Welch ANOVA) and to RANO 3 patients (mean 35.6 ml, SD 19.3; p = 0.009, Welch ANOVA, Fig. 2B). When stratifying patients by the amount of RTV on iMRI (0 ml, ≤1 ml, and >1 ml) we observed a stepwise increase in mean preoperative volumes (5.37 ml, SD 7.17; 9.28 ml, SD 11.3; and 25.4 ml, SD 18.6, respectively). Differences were significant for the >1 ml group on iMRI (p = 0.0004, Welch ANOVA; Fig. 2C). To determine a preoperative tumor volume threshold substantially increasing the probability of a ‘RANO-switch’, we applied a quadratic logistic regression (Youden index), identifying a cut-off of 6.9 ml (Fig. 2D). Altogether, these findings support the association between larger preoperative CE tumor volumes and the likelihood of AR.

**Table 3:** **Logistic regression analysis of predictors for additional resection upon intraoperative MRI (iMRI).** Significant predictors are highlighted in bold. β = regression coefficient; OR = odds ratio; CI = confidence interval; 5-ALA = 5-aminolevulinic acid; IONM = intraoperative neuromonitoring; CR = complete resection; KPS = Karnofsky Performance Status; cont. = continuous.

Significant variables	p-value	HR	95% CI
+ volume post-op			
MGMT	0.047	0.633	0.40 - 0.99
Persistent neurological deterioration	0.002	11.86	2.56 - 54.9
Treatment after re-resection yes/no	<0.001	0.132	0.057 - 0.31
+ RANO 1-2 post-op			
MGMT	0.047	0.633	0.40 - 0.99
Persistent neurological deterioration	0.002	11.86	2.56 - 54.86
Treatment after re-resection yes/no	<0.001	0.132	0.057 - 0.31
+ RTV 0 ml			
MGMT	0.047	0.633	0.40 - 0.99
Persistent neurological deterioration	0.002	11.86	2.56 - 54.86
Treatment after re-resection yes/no	<0.001	0.132	0.057 - 0.31
+ GTR			
MGMT	0.012	0.570	0.37 - 0.88
Persistent neurological deterioration	0.012	7.13	1.55 - 32.8
Treatment after re-resection yes/no	<0.001	0.113	0.048 - 0.26
GTR	0.006	0.497	0.30 - 0.82

### 4.4 Functional integrity is preserved after iMRI-guided resections

We also examined the impact of tumor eloquence on the utility of iMRI and functional outcomes after AR. In 68 patients (45%), tumors involved eloquent areas. RANO class 3 resections were significantly more frequent in eloquently located tumors (74%, n = 17/23), compared to the “RANO-switch” group (49%, n = 20/41) and patients with RANO class 1–2 resections on iMRI (36%, n = 31/86, p = 0.0046; Fig. 2E). The overall rate of new transient neurological deficits was 13% (n = 20), whereas permanent deficits remained in 2% (n = 3). Importantly, there was no statistically significant correlation between AR after iMRI and increased rates of transient (15% with vs. 16% without AR; p = 0.942; Fig. 2F) or permanent deficits (2% vs. 3%; p = 0.5521; Fig. 2F).

### 4.5 Maximized EOR is associated with prolonged survival in patients with recGB

To confirm previous reports of a survival benefit associated with maximized resections, we analyzed patients who underwent re-resection at first recurrence (n = 129). Median survival following re-resection was 11 months (Fig. 2G), without censored patients at the time of analysis. No significant differences were found between CR and CE-RTVs of >0 and ≤ 1 ml (12 vs 10 months; p = 0.3018, log-rank test; Fig. 2G). However, both groups showed significantly improved survival following re-resection compared to RANO class 3 patients (RTV >1 ml, 9 months. p = 0.043 vs > 0-1 ml RTV and p = 0.0078 vs 0 ml RTV; log-rank test, Fig. 2G). The calculated median progression-free survival (PFS) after primary surgery was 10 months in the overall cohort (Table 1): 10 months in the RANO 1–2 group, 8 months in the RANO-switch group, and 8.5 months in the RANO 3 group (Table 2). No significant differences in PFS after first surgery were observed between the subgroups: RANO 1–2 vs. RANO-switch, p = 0.066; RANO 1–2 vs. RANO 3, p = 0.732; or RANO-switch vs. RANO 3, p = 0.347 (log-rank test).

To investigate determinants of survival after re-resection while accounting for potential confounders, we performed multivariable Cox proportional hazards regression analyses including clinical, molecular, and surgical variables. In four separate models, different EOR parameters were assessed: RTV (continuous), RANO categories, absence of any residual CE tumor (RTV 0 ml), and gross total resection (GTR, ≤0.175 ml according to Stummer et al. (2006)). Across the models including RTV, RANO categories, or RTV 0 ml, only MGMT promoter methylation (p = 0.047; HR ∼0.63, Supplementary Fig. 1B), postoperative treatment (p < 0.001; HR ∼0.13, Supplementary Fig. 1C), and persistent neurological deterioration (p = 0.002; HR ∼11.9, Supplementary Fig. 1D) were significantly associated with survival after re-resection, while EOR parameters did not reach significance. In contrast, when GTR (≤0.175 cm^3^) was included as the EOR parameter, it emerged as a significant prognostic factor (p = 0.006; HR 0.50, 95% CI 0.30–0.82, Table 4).

**Table 4:** **Multivariable Cox Proportional Hazards regression analysis of survival after re-resection (n = 100).** Four separate models were tested, each including a different extent-of-resection (EOR) parameter: postoperative tumor volume (continuous), RANO 1–2 postoperatively (≤1 ml), absence of any residual tumor (RTV 0 ml), or gross total resection (GTR, ≤0.175 cm^3^ according to Stummer et al. (2006)). All models included sex, MGMT promoter methylation, age at recurrence, preoperative tumor volume, pre- and postoperative KPS, persistent neurological deterioration, re-resection, and postoperative therapy. Significant variables are reported with p-values, hazard ratios (HR), and 95% confidence intervals (CI). MGMT = O6-methylguanine-DNA methyltransferase; KPS = Karnofsky Performance Status; GTR = gross total resection; RTV = residual tumor volume.

Variable	n = 150 (%)
Sex Male Female	94 (63%) 56 (37%)
Age (years) Mean ± SD Median (IQR)	58.4 (10.4) 59.0 (14.0)
MGMT promoter methylation status at recurrence Methylated Non-methylated Unknown	43 (29%) 78 (52%) 29 (19%)
Stage of disease at the time of re-resection -first recurrence - second recurrence - third recurrence - fourth recurrence	126 (84%) 18 (12%) 5 (3%) 1 (1%)
Time between first and second resection (months) Mean ± SD	15.4 (10.5)
Tumor localization Frontal Temporal Parietal Occipital Insular Deep	43 (28%) 63 (42%) 24 (16%) 16 (11%) 3 (2%) 1 (<1%)
Tumor eloquence	68 (45%)
Intraoperative neuromonitoring±awake craniotomy	18 (12%)
Mean KPS before surgery	83 (10.4)
Mean KPS at discharge	81 (10.8)
Adjuvant chemotherapy after re-resection Temozolomide CCNU/VP-16 BCNU/VM-26 Unknown Bevacizumab Other^a^	24 (16%) 77 (51%) 9 (6%) 4 (3%) 15 (10%) 19 (13%)
Radiotherapy	26 (17%)
No adjuvant treatment	14 (9%)
Median progression-free survival after first resection (months)	10

## 5 Discussion

In this cohort of iMRI-guided resections for recGB, an oncologically meaningful RANO class 1-2 resection (≤1 ml CE-RTV) was attained in 85% of all patients, with 27% showing a threshold-relevant reduction to ≤1 ml CE-RTV between iMRI and epMRI (“RANO-switch”). In 77% of cases, resection was continued after iMRI, with larger preoperative tumor volumes significantly predicting the likelihood of AR. Importantly, transient, or persistent neurological deterioration was not increased in patients with AR. Median survival after re-resection was 11 months across patients with resection at first recurrence and 12 months in patients with no CE-RTV. The aim of our study was not to compare different surgical guidance modalities for tumor resection. Rather, it was designed to highlight the potential clinical value of intraoperative measurement of residual tumor volume in recurrent glioblastoma.

Currently, no standard treatment exists for recGB, and expert opinion is divergent (Weller et al., 2021; van Opijnen et al., 2023). Previous studies identified CR of CE tumor as a key factor for prolonged survival (Weller et al., 2019; Suchorska et al., 2016; Bloch et al., 2012). While iMRI has proven useful in maximizing the EOR for newly diagnosed GB, its role in recGB remains less explored (Wach et al., 2024; Coburger et al., 2017; Shah et al., 2021a). To our knowledge, this is the first larger study specifically examining iMRI in this setting.

An AR rate of 77% emphasizes the utility of iMRI in maximizing the EOR, although an oncologically meaningful “RANO-switch” after AR occurred in only 27%. At the time of iMRI, no CE-RTV had already been achieved in 21% and ≤1 ml CE-RTV in 38% of patients. Nevertheless, all but 3 patients with ≤1 ml RTV on iMRI underwent AR. As RANO Resect criteria suggest similar outcomes for class 1 (0 ml CE + ≤5 ml non-CE) and class 2 (0–1 ml CE ± >5 ml non-CE) resections, many patients already had achieved prognostically favorable thresholds before AR. Notably, in multivariable analysis, only GTR (≤0.175 ml RTV), but not RANO classes, emerged as a significant predictor of survival after re-resection. Although patients undergoing iMRI presented with larger pre-operative tumor volumes and shorter overall post-operative survival, iMRI may still provide a survival benefit in patients with larger tumors, as suggested by the trend toward improved post-operative survival in this subgroup.

Recently, molecularly-guided stratification revealed heterogeneous outcomes across GB methylation subtypes, also at tumor recurrence (Drexler et al., 2024), suggesting future resection thresholds lower than 1 ml may become relevant and justify AR also in cases with as little as 1 ml CE-RTV on iMRI. With growing intraoperative access to molecular data, resections could increasingly be tailored to both functional anatomy and tumor biology. Yet, even under current RANO definitions, iMRI provides critical real-time information. Especially in eloquent locations, iMRI can confirm when RTV ≤1 ml has been reached, supporting decisions to stop resection safely (Drexler et al., 2023; Patel et al., 2025; Djirackor et al., 2021). Comparability or synergy between 5-ALA and iMRI could not be addressed, since 5-ALA, alone or combined with iMRI, was employed in few patients only. 5-ALA is currently seen as non-inferior to iMRI in newly diagnosed GB (Roder et al., 2023), but has been shown to be a viable adjunct at recurrence as well (Nabavi et al., 2009), although concerns over lower specificity and negative predictive value in pretreated patients exist (Broekx et al., 2020; Suero Molina et al., 2019).

Given the logistical demands of iMRI, we assessed predictors for AR, with preoperative tumor volume emerging as the only significant factor. Furthermore, quadratic regression analysis indicated a high probability of RANO-switch when preoperative CE volume exceeded 6.9 ml, suggesting tumor size as a practical criterion for selecting patients who benefit most from iMRI guidance. The observed rate of additional resection (AR) following iMRI acquisition suggests that iMRI was not used merely to confirm the achieved EOR, but rather in situations where the surgeon considered the intended resection endpoint to have been reached or where further tumor removal had to be balanced against functional pertaining to the risk of neurological morbidity. In this context, iMRI frequently provided clinically relevant information that prompted further safe resection, consistent with similarly high rates of additional resection reported in previous glioma surgery series (Senft et al., 2011; Roder et al., 2023; Shah et al., 2021b). Furthermore, variability between surgeons and the evolving institutional experience with iMRI likely influenced the timing of imaging and intraoperative decision-making throughout the study period.

In recGB, eloquent location is associated with reduced survival, likely due to limited resectability (Park et al., 2010). Accordingly, our data showed higher rates of eloquent tumors among RANO class 3 resections. Transient neurological deterioration occurred in 15% of cases and was not significantly increased in patients undergoing AR. In the past, AR has been linked to a higher risk of neurological deterioration by some studies and refuted by others (Stummer et al., 2011). Permanent deficits remained at 2%, lower than previously reported rates of 8–20% in recurrent glioma surgery^,^(Yang et al., 2022)^,^ (Blobner et al., 2023)^-^ (van Opijnen et al., 2024). It is however important to consider the inherent complexity of glioma surgery when interpreting these results, since tumor resection is influenced by multiple factors, including anatomical location, infiltration patterns, and patient-specific variables, which may impact surgical morbidity and outcomes (Ganau et al., 2018). These findings support the safety of maximizing EOR with iMRI.

Median survival after re-resection of 11 months in our cohort aligns with previous reports (Ringel et al., 2016). This outcome may reflect our center's pro-surgical policy, including surgery for patients with less favorable prognostic factors, often to enable trial inclusion or molecular analysis, and hence *post hoc* emphasizing the importance of patient selection (Alhalabi et al., 2024; Blobner et al., 2023; Brennan et al., 2021). Patients of all RANO classes were comparable in confounding factors such as pre- and postoperative KPS, MGMT promoter methylation status and adjuvant therapy. GTR (≤0.175 ml RTV) was the only significant surgical predictor of survival after recurrence.

Limitations of this study include its retrospective, single-center design, and absence of control groups without iMRI. Because of the inclusion criteria of the study, it might not wholly represent the heterogenous landscape of cases with recurrent GB. Findings also rely on prespecified RANO classes, although Cox regression analysis did not confirm the respective RTV cut-offs as predictive of survival, likely due to low numbers of RANO class 3 patients and lack of FLAIR volumetry. As such, RANO classes 1 and 2 were pooled where appropriate. The differing prognostic performance of the applied extent-of-resection thresholds, including the <0.175 ml cutoff and the 0–1 ml classification both previously proposed in the literature (Karschnia et al., 2023; Stummer et al., 2006, 2008), likely reflects methodological differences in threshold definition and cohort distribution rather than a distinct biological effect, and should therefore be interpreted with caution.

Despite these limitations, we present a contemporary cohort of 150 volumetrically analyzed recGB patients, confirming the utility of iMRI-guided re-resection, especially in patients with preoperative CE tumor volumes >6.9 ml. AR after iMRI was safe, with low rates of permanent deficits. While molecularly guided stratification is increasingly important, iMRI combined with intraoperative molecular testing may help individualize surgical strategies.

## 6 Conclusion

Intraoperative MRI provides valuable assessment of residual tumor and facilitates real-time decision-making in recGB surgery. AR significantly increased the rate of RANO class 1–2 resections without added neurological risk. Preoperative CE tumor volume was the only independent predictor for AR upon iMRI endorsing routine iMRI use in recGB particularly for larger tumors. While survival outcomes appeared comparable between RANO class 1 and class 2 resections, these findings should be interpreted with caution given the limited subgroup sizes and the methodological limitations associated with applying volumetric thresholds in the recurrent setting. Nevertheless, the data suggest that achieving ≤1 ml residual contrast-enhancing tumor may represent a clinically relevant surgical goal in selected patients.

## Author contribution

Study concept and design: CJ, OTH, KM. Data collection and analysis: OTH, KM, CJ, NG, KN, MJ, SJ. Data interpretation: OTH, CJ. Writing the manuscript: OTH, CJ. Reviewing and editing: CJ, OTH, MB, SK, PDT, AU. Supervision: CJ. All authors approved the final version of the submitted manuscript.

## Data availability

The datasets generated and/or analyzed during the current study are available from the corresponding author on reasonable request.

## Funding

The authors declare that no funds, grants, or other support were received during the preparation of this manuscript. OTH is financed by the Clinician-Scientist-Program of the Medical Faculty of the Heidelberg University.

## Declaration of competing interest

The authors declare the following financial interests/personal relationships which may be considered as potential competing interests: SMK is a consultant for Brainlab AG, Ulrich Medical, and Need Inc., shareholder of Need Inc. All authors declare that they have no conflict of interest regarding the materials used or the results presented in this study. All authors declare no personal, financial, or institutional interest in any drugs, materials, or devices described in this article. The authors have no further relevant financial or non-financial interests to disclose.

If there are other authors, they declare that they have no known competing financial interests or personal relationships that could have appeared to influence the work reported in this paper.
