# Supplementary material for: The utility of intraoperative magnetic resonance imaging in recurrent glioblastoma surgery: A volumetric analysis
Source: Brain Spine. 2026 Jun 30;6:106135. doi: 10.1016/j.bas.2026.106135 (PMC13356750; doi:10.1016/j.bas.2026.106135)
Supplement: Multimedia component 1 [file mmc1.docx]

**Supplementary Figure S1: A: Exemplary MRI scans of three recGB cases undergoing re-resection, depicting different resection scenarios.** (1) upper panel: no residual CE tumor on intraoperative MRI (iMRI) which is confirmed by epMRI, (2) middle panel: residual CE tumor (>1ml) on iMRI which undergoes additional resection, yielding a “RANO-switch” from a class 3 to a class 1-2 resection. Noe the annotated tumor and choroid plexus. (3) lower panel: residual CE tumor of <1ml on iMRI which undergoes additional resection, yielding a RANO class 1 resection. preopMRI= preoperative MRI. **B-D:** Kaplan–Meier survival curves following surgery at first recurrence, stratified by different variables. Log-rank (Mantel-Cox) test was used with p-values embedded in Figure. **B:** Kaplan-Meier survival curves of the final MRI cohort (n=150) showing a median survival after re-resection of 354 days). **C:** Distribution of postoperative resection outcomes according to residual tumor volume (RTV) thresholds and RANO resection classes. Stacked bar plots illustrate the proportion of patients with RTV cutoffs of <0.175 ml versus >0.175 ml, complete resection (0 ml) versus residual tumor (>0 ml), and RTV <1 ml versus >1 ml (corresponding RANO 1/2 or RANO 3). Numbers within bars indicate patient counts with corresponding percentages.
